# The ZmbHLH47-ZmSnRK2.9 Module Promotes Drought Tolerance in Maize

**DOI:** 10.3390/ijms25094957

**Published:** 2024-05-01

**Authors:** Zhenwei Yan, Fajun Zhang, Chunhua Mu, Changle Ma, Guoqi Yao, Yue Sun, Jing Hou, Bingying Leng, Xia Liu

**Affiliations:** 1 Maize Research Institute, Shandong Academy of Agricultural Sciences, Jinan 250100, China; yanzwplant@sina.com (Z.Y.); zhangfj2009@163.com (F.Z.); maizesd@163.com (C.M.); yaoguoqi927@sohu.com (G.Y.); 2 College of Life Sciences, Shandong Normal University, Jinan 250300, China; machangle@sdnu.edu.cn; 3 College of Agronomy, Qingdao Agricultural University, Qingdao 266109, China; sunyue3070601@163.com; 4 School of Agriculture, Ludong University, Yantai 264001, China; houjing@m.ldu.edu.cn

**Keywords:** ABA response, drought tolerance, maize, ZmbHLH47, ZmSnRK2.9

## Abstract

Drought stress globally poses a significant threat to maize (*Zea mays* L.) productivity and the underlying molecular mechanisms of drought tolerance remain elusive. In this study, we characterized ZmbHLH47, a basic helix–loop–helix (bHLH) transcription factor, as a positive regulator of drought tolerance in maize. *ZmbHLH47* expression was notably induced by both drought stress and abscisic acid (ABA). Transgenic plants overexpressing *ZmbHLH47* displayed elevated drought tolerance and ABA responsiveness, while the *zmbhlh47* mutant exhibited increased drought sensitivity and reduced ABA sensitivity. Mechanistically, it was revealed that ZmbHLH47 could directly bind to the promoter of *ZmSnRK2.9* gene, a member of the subgroup III SnRK2 kinases, activating its expression. Furthermore, *ZmSnRK2.9*-overexpressing plants exhibited enhanced ABA sensitivity and drought tolerance, whereas the *zmsnrk2.9* mutant displayed a decreased sensitivity to both. Notably, overexpressing *ZmbHLH47* in the *zmsnrk2.9* mutant closely resembled the *zmsnrk2.9* mutant, indicating the importance of the ZmbHLH47-ZmSnRK2.9 module in ABA response and drought tolerance. These findings provided valuable insights and a potential genetic resource for enhancing the environmental adaptability of maize.

## 1. Introduction

Drought stress stands as a prominent environmental factor hindering agricultural progress and global crop productivity [1,2,3,4,5,6]. Insufficient water availability limits plant growth and development, leading to reduced plant production and the substantial impairment of crop performance [7]. In response to drought stress, plants undergo morphological and molecular alterations, manifesting as noticeable changes in photosynthesis, stomatal aperture, hormone level, leaf growth, stem extension, root proliferation, and hydraulic conductivity [7,8,9,10,11]. Maize, a crucial food and industrial crop globally [12,13], faces an escalating threat in its cultivation due to climate changes and unpredictable rainfall patterns, contributing to the increased occurrence of severe drought stress [14]. Furthermore, it is noteworthy that due to its shallow roots, maize exhibits insensitivity to drought and intolerance to soils with water deficiency [15]. Hence, revealing the molecular mechanisms involved in conferring resistance to drought in maize holds significant promise for advancing the breeding and cultivation of drought–tolerant varieties.

The basic helix–loop–helix (bHLH) transcription factors (TFs) constitute the second–largest gene family of TFs in plants [16]. In this family, members exhibit a remarkably conserved bHLH domain, playing a pivotal role in DNA binding and facilitating protein–protein interactions [17,18]. In the maize genome, a total of 208 bHLH genes have been identified [19]. However, only a limited number of bHLH TFs have been recognized for their involvement in conferring drought tolerance in maize. For instance, Gao et al. demonstrated that overexpressing *ZmPIF3* in rice (Oryza sativa) enhanced tolerance to drought and salt stresses [20]. Additionally, ZmPTF1, known for its roles in root development and low–phosphate stress tolerance in maize, was found to improve drought tolerance by promoting ABA synthesis [21]. Moreover, ZmbHLH124 emerged as a positive regulator of drought tolerance in maize, partly due to the direct activation of *ZmDREB2A*, mediating plant responses to water deficit [22]. Moreover, when overexpressed in rice (Oryza sativa), ZmPIF1 has demonstrated drought resistance, possibly linked to reduced stomatal aperture and transpiration rate [23]. However, a more comprehensive characterization is needed to fully understand the functions and regulatory roles of bHLH TFs associated with maize drought tolerance.

Abscisic acid (ABA) plays a vital role as a phytohormone in plant stress responses [24,25,26,27,28]. Osmotic stress induced by drought triggers a swift accumulation of ABA in plants [29,30]. ABA signals are detected by ABA receptors, initiating downstream signal transduction. In *Arabidopsis*, these receptors comprise the PYRABACTIN RESISTANCE 1 (PYR1), PYR1–like proteins (PYL), or members of the regulatory components of the ABA receptor (RCAR) family of START proteins [31,32,33]. In the absence of ABA, clade A PP2C phosphatases (PP2C–A) establish interactions with and catalyzes the dephosphorylation of three group III SUCROSE NONFERMENTING1–RELATED SUBFAMILY 2 (SnRK2) protein kinases (SnRK2.2, SnRK2.3, and SnRK2.6/OST1). The accumulation of ABA triggers the binding between PYR/PYL/RCAR receptors and clade A PP2C phosphatases (PP2C–A). Consequently, SnRK2 kinases are released and activated, leading to the phosphorylation and activation of downstream ABA–responsive elements–binding protein/ABA–responsive elements–binding factor (AREB/ABF) proteins [34,35,36,37]. Through manipulating stomatal closure and water loss, the ABA signaling transduction pathway confers drought tolerance in plants [38]. The maize genome contains 13 genes encoding PYR/PYL/RCAR ABA receptors [39], 13 genes encoding clade A PP2C phosphatases [40], and 14 genes encoding SnRK2 kinases [41]. Recently, several studies have uncovered the essential roles of some *ZmPYL* or *ZmPP2C-A* genes in regulating plant drought response. For example, He et al. found that transgenic plants overexpressing *ZmPYL8*, *ZmPYL9*, and *ZmPYL12* were more tolerant to drought stress, which were associated with the accumulation of proline and the enhanced expression of drought-related marker genes [39]. In addition, members of maize clade A PP2C phosphatases, *ZmPP2C-A2*, *ZmPP2C-A6*, and *ZmPP2C-A10*, were reported to negatively regulate drought tolerance by mediating ABA signaling [42]. However, despite their importance, the function of *ZmSnRK2* genes in the regulation of maize drought tolerance remains to be elucidated.

In this contemporary investigation, it was attempted to delineate the affirmative contributions of the bHLH transcription factor ZmbHLH47 and subgroup III SnRK2 protein kinase ZmSnRK2.9 in the ABA response and drought tolerance in maize. Moreover, ZmbHLH47 has been recognized as a factor that could directly bind to the *ZmSnRK2.9* promoter, leading to the activation of its expression and finely tuning the levels of ABA–responsive genes, particularly under a drought stress condition. The research contributes to a comprehensive comprehension of the mechanisms that would underlie drought stress tolerance in maize and provides a valuable genetic asset for the advancement of drought–resistant maize varieties.

## 2. Results

### 2.1. Identifying and Analyzing the Sequence of ZmbHLH47

In the pursuit of identifying novel abiotic stress–tolerant genes, we conducted an analysis of publicly available expression data from a previously published study [43]. The investigation hinted at the potential candidacy of ZmbHLH47 (GRMZM2G133675) as a novel abiotic stress–responsive gene. The ZmbHLH47 gene (GRMZM2G133675) encompasses a complete open reading frame (ORF) spanning 825 base pairs, encoding a polypeptide of 274 amino acid residues. This polypeptide has a molecular mass of 29.36 kilodalton (kDa) and an isoelectric point (pI) of 5.65. A phylogenetic tree, constructed based on ZmbHLH47 and its homologs in maize, rice, and *Arabidopsis*, revealed a close relationship among ZmbHLH47, OsbHLH1, OsbHLH63, and AtPOPEYE (Figure S1A). A precise analysis involved aligning the amino acid sequences of ZmbHLH47 with those of its homologous proteins, as illustrated in Figure S1B. The results underscored that the ZmbHLH47 protein is a canonical bHLH transcription factor, which is characterized by a conserved bHLH domain known for its role in DNA binding and the formation of protein complexes [44,45,46].

### 2.2. ZmbHLH47 Functions as a Transcription Factor

To assess the transcription factor (TF) activity of ZmbHLH47 and pinpoint the region responsible for this activity, distinct segments of the coding sequence were autonomously linked to the GAL4 DNA–binding domain in the *pGBKT7* vector. These segments encompassed the entire coding sequence (1–274 aa), the N–terminal and middle domain sequence (1–127 aa), the N–terminal sequence (1–67 aa), the middle domain and C–terminal sequence (68–274 aa), and the C–terminal sequence (128–274 aa). Yeast cells transformed with ZmbHLH47 (1–274 aa), ZmbHLH47 (68–274 aa), and ZmbHLH47 (128–274 aa) displayed a robust growth on SD/–Trp/–His/–Ade medium (Figure S2A), unequivocally indicating the transcriptional activation activity of ZmbHLH47. Notably, the C–terminal region (128–274 aa) was identified as a contributor to the transactivation activity of ZmbHLH47.

Furthermore, to delineate the subcellular localization of ZmbHLH47, we carried out transient co–expression assays by introducing a construct encoding GFP–tagged ZmbHLH47 and a nuclear marker (D53–mCherry) into maize protoplasts. Notably, intense green fluorescence emitted by the ZmbHLH47–GFP protein was distinctly confined to the nucleus, displaying accurate co–localization with the established nuclear marker D53–mCherry protein [47]. In contrast, the GFP protein signal was discerned throughout the plasma membrane, cytoplasm, and nucleus, presenting a stark disparity, highlighting distinct cellular compartments (Figure S2B,C). These findings consistently supported the role of ZmbHLH47 as a transcription factor, as its protein predominantly localizes in the nucleus.

### 2.3. Expression Analysis of ZmbHLH47

To ascertain the tissue–specific transcript abundance of *ZmbHLH47* in maize, we undertook quantitative reverse transcription polymerase chain reaction (qRT–PCR) analysis. The outcomes revealed a noticeable pattern of expression for *ZmbHLH47*, with the apex in leaves, succeeded by descending levels in roots, embryos, endosperms, pollens, and stems. Conversely, relatively diminished expression levels were noted in ears and silks (Figure S3A). Furthermore, it was attempted to carry out qRT–PCR to explore the relative expression level of *ZmbHLH47* under abiotic stress and exogenous ABA. Intriguingly, treatments with NaCl, PEG, and exogenous ABA swiftly and significantly induced the expression level of *ZmbHLH47* (Figure S3B–D). These outcomes strongly suggested that ZmbHLH47 could potentially play a notable function in mediating the maize response to diverse abiotic stresses.

### 2.4. ZmbHLH47 Functions as a Positive Regulator of Drought Tolerance in Maize

**Figure 1:**
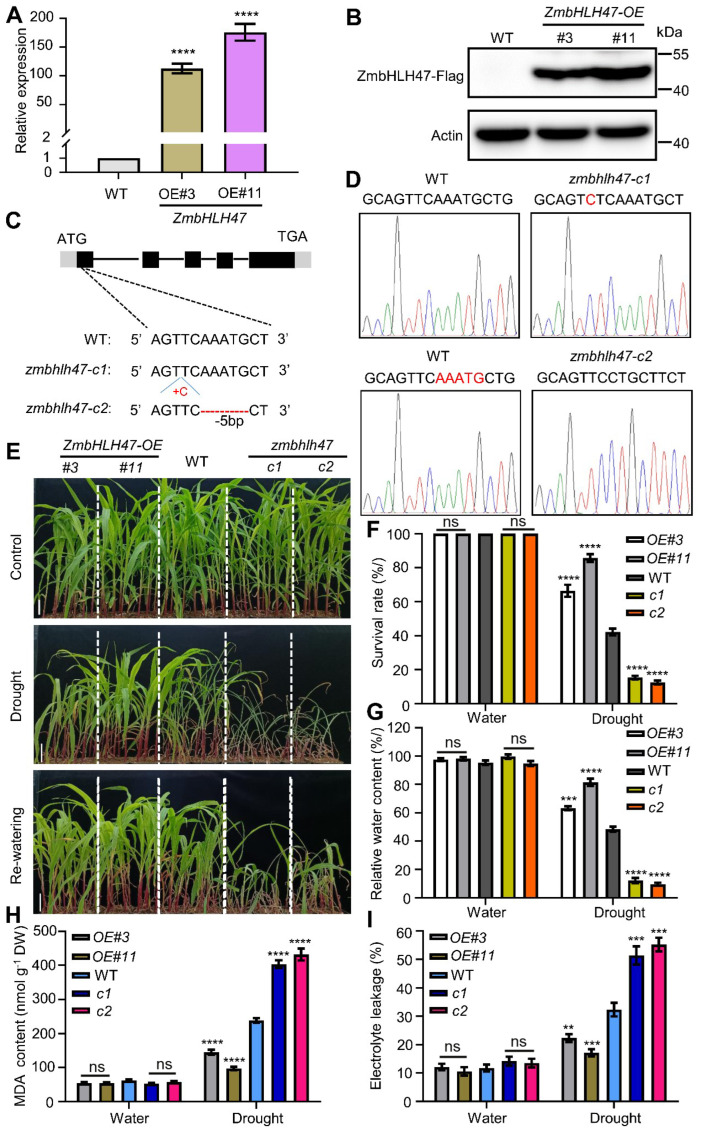
ZmbHLH47 confers drought tolerance in maize. (**A**) The transcript abundance of *ZmbHLH47* in WT and *ZmbHLH47-OE* seedlings. Expression in WT was set to 1.00. Data shown are means ± SD of three biological replicates. **** indicates significant difference to the corresponding controls with *p* < 0.0001 (Student’s *t*−test). (**B**) The protein abundance of ZmbHLH47 in *ZmbHLH47-OE* transgenic seedlings (#3, #11). Anti–Flag antibody was utilized to detect ZmbHLH47. Actin served as a control. (**C**) Schematic diagrams of *zmbhlh47-crispr* mutants generated using CRISPR/Cas9–mediated genome editing. (**D**) The *zmbhlh47-c1* and *zmbhlh47-c2* mutations were identified using Sanger sequencing compared with WT. (**E**) Drought tolerance phenotypes of WT, *ZmbHLH47-OE*, and *zmbhlh47-crispr* plants. Twelve−day−old seedlings were subjected to drought stress by withholding water for 8 days, after which they were re–watered for 3 d (Re−watering). Scale bars = 5 cm. (**F**) Statistical analysis of survival rates after drought stress as shown in (**E**). At least 30 seedlings of each line per replicate were used for survival rate analysis. Data shown are means ± SD of three biological replicates. ns indicates no significant difference to the corresponding controls. **** indicates significant difference to the corresponding controls with *p* < 0.0001 (Student’s *t*−test). (**G**) Relative water content (RWC) in WT, *ZmbHLH47-OE*, and *zmbhlh47-crispr* plants under well−watered and drought conditions. Data shown are means ± SD of three biological replicates. ns indicates no significant difference to the corresponding controls. Significant differences are indicated using Student’s *t*−test: *** *p* < 0.001; **** *p* < 0.0001. (**H**,**I**) Malondialdehyde (MDA) content (**H**) and the percentage leakage of electrolyte (**I**) of WT, *ZmbHLH47-OE*, and *zmbhlh47-crispr* plants under well–watered and drought conditions. DW, dry weight. The values are presented as means ± SD of three biological replicates. ns indicates no significant difference to the corresponding controls. ** *p* < 0.01; *** *p* < 0.001; **** *p* < 0.0001, indicating significant differences to the corresponding controls (Student’s *t*−test).

To elucidate the functional role of ZmbHLH47 in maize, it was attempted to engineer both the overexpression lines and loss–of–function mutants. Post-examining the transcript level and protein abundance, we judiciously selected two independent overexpression lines (*ZmbHLH47-OE#3* and *ZmbHLH47-OE#11*) for detailed investigation (Figure 1A,B). Employing the Clustered Regularly Interspaced Short Palindromic Repeats (CRISPR) associated 9 (CRISPR/Cas9) genome editing system [48,49], an attempt was made to establish two loss–of–function mutants of *ZmbHLH47*, designated as *zmbhlh47-c1* and *zmbhlh47-c2*. *zmbhlh47-c1* harbored a 1–bp insertion (42–bp downstream of ATG), while *zmbhlh47-c2* carried a 5–bp deletion (from 44– to 50– bp downstream of ATG), inducing frameshifts in the ORF and the premature termination of translation (Figure 1C,D).

The *ZmbHLH47-OE* lines, *zmbhlh47-crispr* mutants, and wild−type (WT) plants were cultivated until they reached a 12−day−old stage in soil. Subsequently, they underwent 8 days of drought stress without watering, followed by 3 days of rewatering. Notably, no discernible differences in growth phenotypes were discerned among WT, *ZmbHLH47-OE*, and *zmbhlh47-crispr* plants under well-watered conditions (Figure 1E). In contrast, under conditions of drought stress or rehydration, *ZmbHLH47-OE* plants exhibited less severe wilting, whereas *zmbhlh47-crispr* plants incurred more damage compared with WT plants (Figure 1E). In concurrence with these observations, after drought stress, *ZmbHLH47-OE* plants displayed a significantly higher survival rate, and their leaf relative water content was significantly greater, while *zmbhlh47-crispr* plants exhibited significantly lower values relative to WT plants (Figure 1F,G).

In addition, as malondialdehyde (MDA) content and electrolyte leakage serve as indicative markers of drought tolerance [50,51], it was attempted to calculate these parameters for WT, *ZmbHLH47-OE*, and *zmbhlh47-crispr* plants. Prior to drought stress, MDA content and electrolyte leakage exhibited similar levels among WT, OE, and Crispr lines (Figure 1H,I). However, subsequent to drought stress, compared with those of WT plants, the MDA content and electrolyte leakage were dramatically lower in *ZmbHLH47-OE* plants and significantly higher in *zmbhlh47-crispr* plants, respectively (Figure 1H,I). These outcomes strongly advocated for ZmbHLH47 as a positive regulator of drought tolerance in maize.

Furthermore, we extended our inquiry to assess the involvement of ZmbHLH47 in the response to salt stress. The seedlings of WT, *ZmbHLH47-OE*, and *zmbhlh47-crispr* were cultivated until they reached a two−week−old stage and were then subjected to either water (control) or 200 mM NaCl treatment. Intriguingly, in terms of growth performance, dry weight, and survival rate, no overt distinctions were discerned among WT, *ZmbHLH47-OE*, and *zmbhlh47-crispr* plants under both normal and salt stress conditions (200 mM NaCl) (Figure S4A–C). This unequivocally suggested that ZmbHLH47 does not play a role in the salt stress response in maize. Hence, based on the aforementioned findings, ZmbHLH47 emerges as a discernible positive regulator specifically implicated in maize drought tolerance, while remaining unrelated to salt tolerance.

### 2.5. ZmbHLH47 Promotes ABA Sensitivity in Maize

**Figure 2:**
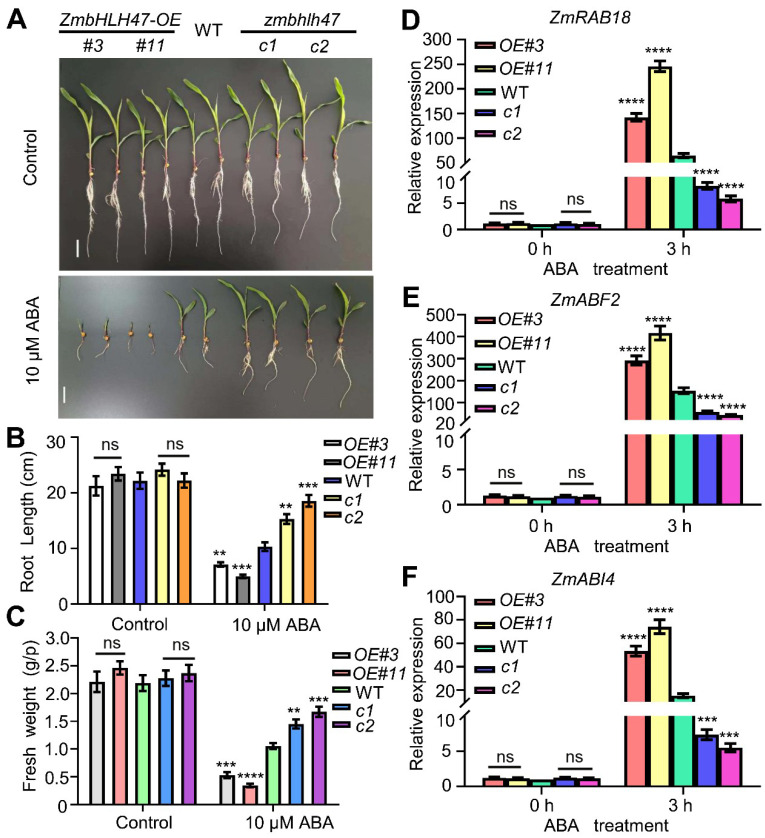
ZmbHLH47 enhances ABA sensitivity and promotes ABA response in maize. (**A**) Growth performance of WT, *ZmbHLH47-OE*, and *zmbhlh47-crispr* plants grown in Hoagland solution with or without ABA. (**B**,**C**) The root lengths and fresh weights of WT, *ZmbHLH47-OE*, and *zmbhlh47-crispr* plants treated with 0 or 10 µM ABA for 7 d. Data are means of three biological replicates means ± SD (*n* = 60 for (**B**,**C**)). ns indicates no significant difference to the corresponding controls. ** *p* < 0.01; *** *p* < 0.001; **** *p* < 0.0001, indicating significant differences to the corresponding controls (Student’s *t*−test). (**D**–**F**) Determination of ABA–responsive gene expression levels among WT, *ZmbHLH47-OE*, and *zmbhlh47-crispr* plants in response to treatment with 50 µM ABA. Expression in the untreated WT was set to 1.00. Data shown are means ± SD of three biological replicates. ns indicates no significant difference to the corresponding controls. Significant differences are indicated using Student’s *t*−test: *** *p* < 0.001; **** *p* < 0.0001.

ABA is widely acknowledged for its pivotal role in plant-drought resistance [52,53,54]. In an endeavor to further elucidate the involvement of ZmbHLH47 in ABA signaling, we compared the phenotypes of various genotypes during the seedling growth stage. The outcomes revealed that, concerning root length and fresh weight, no significant differences were discerned among the overexpression lines, WT, and the mutants under a normal condition. In contrast, when subjected to 10 µM ABA treatment, *ZmbHLH47* overexpression lines exhibited elevated sensitivity to ABA, while the mutants displayed greater ABA insensitivity relative to the WT control (Figure 2A–C).

Moreover, qRT−PCR analysis was undertaken to quantify the transcript levels of selected ABA−responsive genes, namely *ZmRAB18*, *ZmABF2*, and *ZmABI4* [55,56,57,58]. Under normal conditions, all three genes exhibited similar expression levels across different genotypes. However, following ABA treatment, their expression levels were markedly elevated in the overexpression lines and significantly reduced in the mutants compared to WT plants, respectively (Figure 2D–F). Collectively, these findings robustly underscored that ZmbHLH47 exerts a positive influence on ABA response, which may likely contribute to its role in enhancing drought tolerance in maize.

### 2.6. ZmbHLH47 Specially Regulates the Expression of ZmSnRK2.9

To further unravel the molecular mechanisms underlying ZmbHLH47′s role as a positive regulator in maize drought tolerance, we investigated whether it modulates the ABA biosynthesis or downstream signal transduction pathway under drought stress condition. Intriguingly, as depicted in Figure S5A–D, no significant differences were discerned among WT, *ZmbHLH47-OE*, and *zmbhlh47-crispr* plants under both normal and drought conditions, upon the evaluation of the ABA content and the expression levels of pivotal genes involved in ABA biosynthesis. It was unveiled that ABA biosynthesis was not intricately associated with ZmbHLH47. Furthermore, an assessment of the expression levels of genes related to ABA signal transduction, including *ZmPYLs*, *ZmPP2C-As*, and *ZmSnRK2s* [39,40,41], revealed through qRT−PCR results that *ZmSnRK2.9*, a member of subgroup III SnRK2 kinases [41], demonstrated an elevated level of induction in *ZmbHLH47*–overexpressing plants versus wild−type plants following drought stress treatment (Figures S6A–K, S7A–J and S8A–K). Conversely, the degree of induction in *zmbhlh47-crispr* plants was significantly lower than that in WT plants (Figures S6A–K, S7A–J and S8A–K). In concert, these outcomes suggested that ZmbHLH47 selectively regulates the expression of *ZmSnRK2.9*, contributing to its positive role in drought tolerance.

### 2.7. ZmSnRK2.9 Is a Direct Target of ZmbHLH47

**Figure 3:**
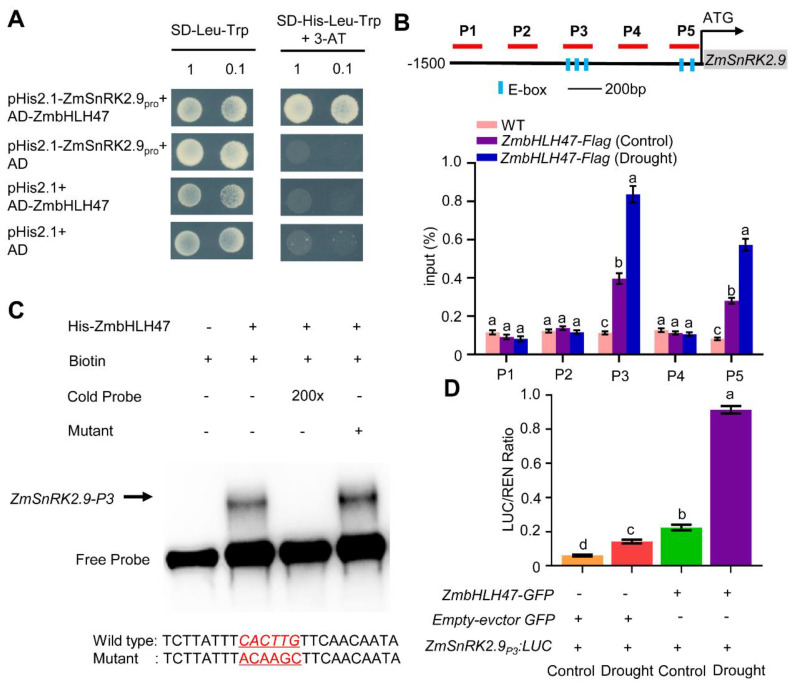
ZmbHLH47 directly binds to the *ZmSnRK2.9* promoter and activates its expression. (**A**) Y1H assay showing direct binding of ZmbHLH47 to the *ZmSnRK2.9* promoter in yeast. AD: *pGADT7*, −WL: −Leu−Trp, −WLH + 3AT: −His−Leu−Trp + 3−Amino−1,2,4−triazole. (**B**) ChIP assay showing the binding of ZmbHLH47 to the *ZmSnRK2.9* promoter in vivo. *ZmbHLH47-Flag* seedlings were conducted with sufficient water or drought for 12 h. Utilizing specific primers (P1−P5) within the *ZmSnRK2.9* promoter region, qPCR was performed to quantify the immunoprecipitated DNA. Input (%) represents relative enrichment. Data shown are means ± SD of three biological replicates with one−way ANOVA and Tukey’s test. Different letters indicate significant differences (*p* < 0.05). (**C**) EMSA assay showing ZmbHLH47 specifically binds to the E−box in the P3 fragment of *ZmSnRK2.9* promoter. Wild-type and mutated competitor sequences are illustrated at the bottom. Arrowhead represents the shifted band. Three independent repeats showed similar results. (**D**) Dual−LUC assay showing that ZmbHLH47 positively regulates *ZmSnRK2.9* transcription in maize protoplasts. Protoplasts co−transformed with *ZmbHLH47-GFP* and *pZmSnRK2.9:LUC* were treated with or without 50 mM mannitol for 1 h. REN represents Renilla LUC, LUC represents firefly LUC. Data shown are means ± SD of three biological replicates with one−way ANOVA and Tukey’s test. Different letters indicate significant differences (*p* < 0.05).

To further figure out whether ZmbHLH47 directly modulates the expression of *ZmSnRK2.9*, we attempted to evaluate ZmbHLH47′s binding capacity to the *ZmSnRK2.9* promoter using yeast one−hybrid (Y1H) assay. As depicted in Figure 3A, yeast cells, subjected to co−transfection with the *pHis2.1-ZmSnRK2.9_pro_* and the *pGADT7-ZmbHLH47* plasmids, demonstrated an unhampered growth on SD/−His−Leu−Trp medium containing 3–AT. This outcome highlighted the plausible interaction of *ZmbHLH47* with the promoter region of *ZmSnRK2.9* within the yeast system.

The bHLH domain in bHLH–type transcription factors exhibited a preference for binding E–box (CANNTG) motifs in downstream target genes [45,59,60]. Multiple E−boxes were identified in the *ZmSnRK2.9* promoter region, hinting at *ZmSnRK2.9* being a potential target of ZmbHLH47 (Figure 3B). To verify this hypothesis, we conducted chromatin immunoprecipitation (ChIP) assay using an anti−Flag antibody with 14−day−old *ZmbHLH47-OE* seedlings, both under normal conditions and following a 12 h drought treatment, followed using qPCR. Out of the identified five putative binding sites (P1–P5) within the *ZmSnRK2.9* promoter, ChIP−qPCR analysis unveiled that ZmbHLH47–Flag exhibited specific binding affinity to the P3 and P5 sites in *ZmbHLH47-Flag* samples, highlighting a distinction absence in the WT ChIP samples (Figure 3B). The significant elevation in fold enrichment at the P3 and P5 sites following drought treatment is noteworthy, underscoring an augmented binding affinity of ZmbHLH47 to the *ZmSnRK2.9* promoter in response to drought conditions (Figure 3B). Subsequent electrophoretic mobility shift assays (EMSAs) provided evidence that His−ZmbHLH47 could directly interact with the conserved P3 motif within the *ZmSnRK2.9* promoter (Figure 3C). The incremental inclusion of an unlabeled wild−type probe effectively disrupted ZmbHLH47 binding to the biotin–labeled probe, while the mutated probe proved ineffective in competing for binding (Figure 3C). These outcomes unequivocally highlighted that ZmbHLH47 could directly interact with the *ZmSnRK2.9* promoter.

Additionally, transitory transactivation assessment was undertaken employing the P3 promoter fragment of *ZmSnRK2.9* in a dual luciferase (LUC) reporter system (Figure 3D). Maize leaf protoplasts underwent co−transformation with the *ZmbHLH47-GFP* effector construct and the *ZmSnRK2.9_P3_:LUC* reporter construct. Consistent with expectations, drought treatment elicited the anticipated induction of *ZmSnRK2.9* transcription. Furthermore, the co−transformation of the LUC reporter with ZmbHLH47–GFP led to the activation of *ZmSnRK2.9* expression under normal conditions, and this activation was even more pronounced after drought treatment (Figure 3D). Hence, these outcomes suggested that ZmbHLH47 positively regulates *ZmSnRK2.9* transcription by directly binding to its promoter.

### 2.8. The Promotive Function of ZmSnRK2.9 in Drought Tolerance in Maize

**Figure 4:**
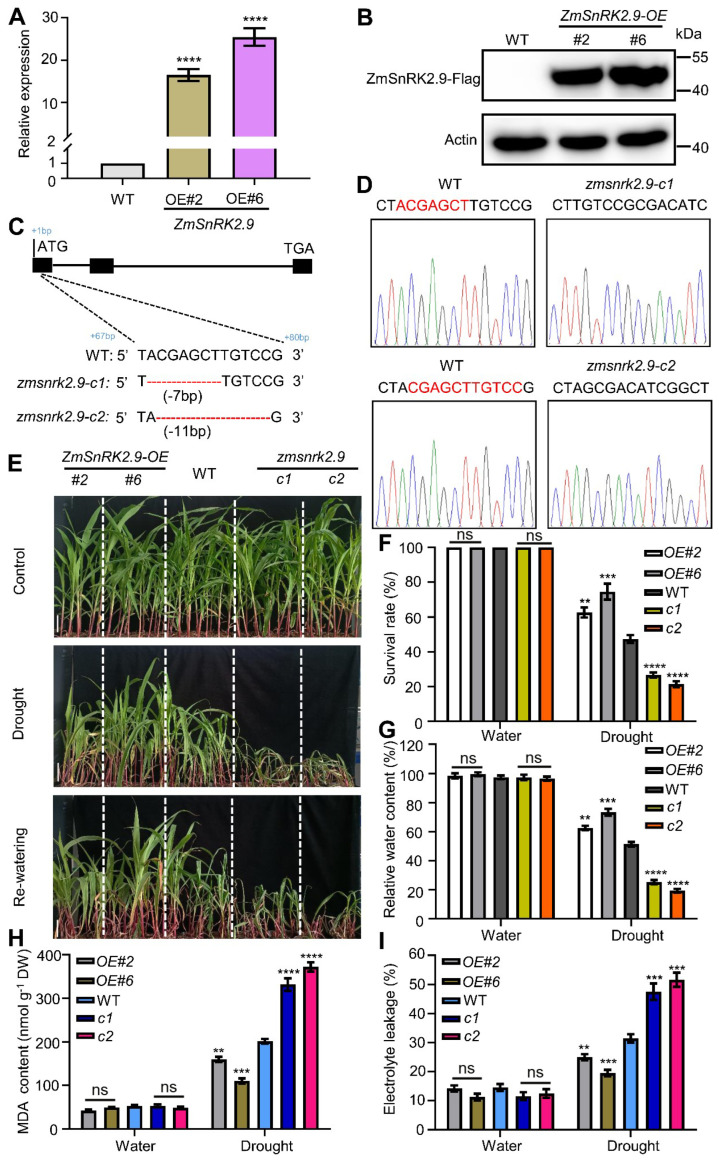
ZmSnRK2.9 is a positive regulator of drought tolerance in maize. (**A**) The transcript abundance of *ZmSnRK2.9* in WT and *ZmSnRK2.9-OE* seedlings. Expression in WT was set to 1.00. Data shown are means ± SD of three biological replicates. **** indicates significant difference to the corresponding controls with *p* < 0.0001 (Student’s *t*−test). (**B**) The protein abundance of ZmSnRK2.9 in *ZmSnRK2.9-OE* transgenic seedlings (#2, #6). Anti–Flag antibody was utilized to detect ZmSnRK2.9. Actin served as a control. (**C**) Schematic diagrams of *zmsnrk2.9-crispr* mutants generated using CRISPR/Cas9−mediated genome editing. (**D**) The *zmsnrk2.9-c1* and *zmsnrk2.9-c2* mutations were identified using Sanger sequencing compared with WT. (**E**) Drought tolerance phenotypes of WT, *ZmSnRK2.9-OE*, and *zmsnrk2.9-crispr* plants. Twelve−day−old seedlings were subjected to drought stress by withholding water for 8 days, after which they were re–watered for 3 d (Re−watering). Scale bars = 5 cm. (**F**) Statistical analysis of survival rates after drought stress as shown in (**E**). At least 30 seedlings of each line per replicate were used for survival rate analysis. Data shown are means ± SD of three biological replicates. ns indicates no significant difference to the corresponding controls. ** *p* < 0.01; *** *p* < 0.001; **** *p* < 0.0001, indicating significant differences to the corresponding controls (Student’s *t*−test). (**G**) Relative water content (RWC) in WT, *ZmSnRK2.9-OE*, and *zmsnrk2.9-crispr* plants under well–watered and drought conditions. Data shown are means ± SD of three biological replicates. ns indicates no significant difference to the corresponding controls. Significant differences are indicated using Student’s *t*−test: ** *p* < 0.01; *** *p* < 0.001; **** *p* < 0.0001. (**H**,**I**) Malondialdehyde (MDA) content (**H**) and percentage leakage of electrolyte (**I**) of WT, *ZmSnRK2.9-OE*, and *zmsnrk2.9-crispr* plants under well−watered and drought conditions. DW, dry weight. The values are presented as means ± SD of three biological replicates. ns indicates no significant difference to the corresponding controls. ** *p* < 0.01; *** *p* < 0.001; **** *p* < 0.0001, indicating significant differences to the corresponding controls (Student’s *t*−test).

Subsequently, we assessed whether *ZmSnRK2.9* could play a role in regulating maize drought tolerance. To investigate this, *ZmSnRK2.9*−overexpressing lines (*ZmSnRK2.9-OE#2* and *ZmSnRK2.9-OE#6*) were generated and verified through qRT−PCR and immunoblotting assays (Figure 4A,B). The RNA–guided CRISPR/Cas9 system was employed to generate a loss−of−function mutant of *ZmSnRK2.9* [48]. Two independent CRISPR/Cas9−edited mutants, *zmsnrk2.9-c1* with a 7−bp deletion (from 68 to 74 bp after the ATG start codon) and *zmsnrk2.9-c2* with an 11–bp deletion between nucleotides 69 and 79 after the start codon, were selected for subsequent analysis. Both mutations induced frameshifts and an early termination of translation (Figure 4C,D).

To assess drought tolerance, 12−day−old plants of WT, *ZmSnRK2.9-OE*, and *zmsnrk2.9-crispr* underwent water deficit treatment, followed by a rehydration process, as previously described. As illustrated in Figure 4E, under well−watered conditions, the growth performance of WT, *ZmSnRK2.9-OE*, and *zmsnrk2.9-crispr* plants showed no discernible differences. Conversely, under water deficit and rehydration conditions, the Crispr plants exhibited more severe dehydration, whereas the OE plants displayed less rolling and wilting compared with the WT plants. As anticipated, after drought treatment, OE plants displayed an escalated survival rate and relative water content, whereas Crispr plants manifested a significantly diminished survival rate and relative water content versus WT plants (Figure 4F,G). Additionally, the MDA content and electrolyte leakage were correlated with the growth phenotype, survival rate, and relative water content. Water deficit treatment induced notable increases in the MDA content and electrolyte leakage compared with the well−watered condition in WT plants, and these effects were exacerbated in *zmsnrk2.9-crispr* plants while alleviated in *ZmSnRK2.9-OE* plants (Figure 4H,I). In conclusion, these outcomes highlighted that ZmSnRK2.9 could function as an affirmative modulator of drought tolerance in maize, mirroring the role of ZmbHLH47.

### 2.9. ZmSnRK2.9 Positively Regulates the ABA Response

**Figure 5:**
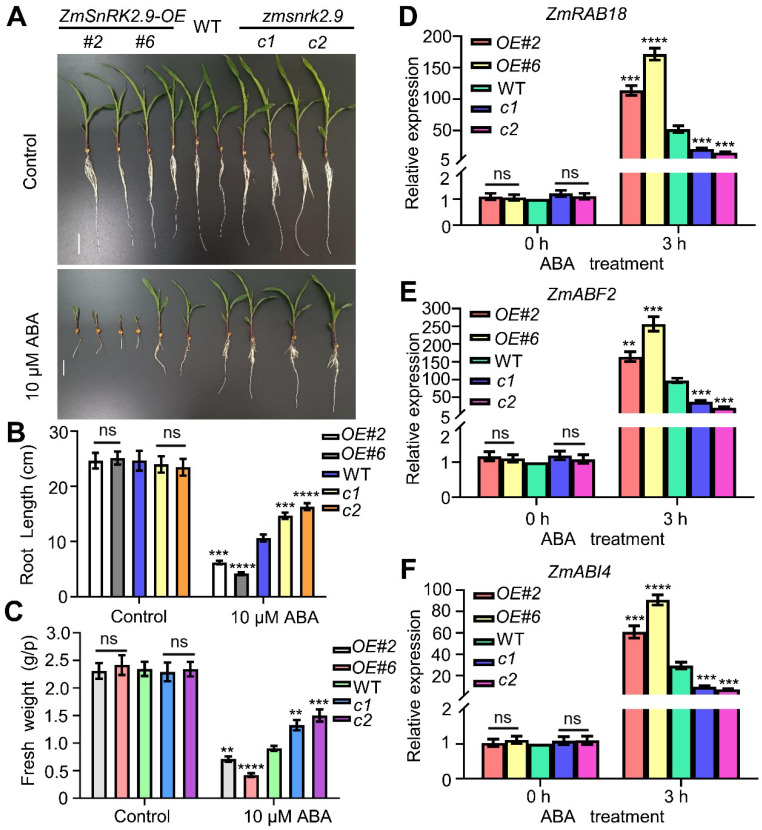
ZmSnRK2.9 positively regulates the ABA response in maize. (**A**) Phenotypes of WT, *ZmSnRK2.9-OE*, and *zmsnrk2.9-crispr* plants grown under control and 10 µM ABA treatment for 8 d. (**B**,**C**) Bar graphs of the root length and fresh weight in (**A**). Data are means of three biological replicates means ± SD (*n* = 60 for (**B**,**C**)). ns indicates no significant difference to the corresponding controls. ** *p* < 0.01; *** *p* < 0.001; **** *p* < 0.0001, indicating significant differences to the corresponding controls (Student’s *t*−test). (**D**–**F**) The expression levels of ABA−responsive genes in WT, *ZmSnRK2.9-OE*, and *zmsnrk2.9-crispr* plants under control and 50 µM ABA treatment. Expression in the untreated WT was set to 1.00. Data shown are means ± SD of three biological replicates. ns indicates no significant difference to the corresponding controls. Significant differences are indicated using Student’s *t*−test: ** *p* < 0.05; *** *p* < 0.001; **** *p* < 0.0001.

To determine the involvement of ZmSnRK2.9 in the ABA−response pathway, we assessed the responses of WT plants, *ZmSnRK2.9-OE*, and *zmsnrk2.9-crispr* plants to different concentrations of ABA (0 and 10 µM). Under the 10 µM ABA treatment, the *zmsnrk2.9-crispr* plants exhibited significantly increased root length and fresh weight compared with the WT, while the *ZmSnRK2.9-OE* plants displayed a shorter root length and smaller fresh weight (Figure 5A–C). Additionally, we examined the expression levels of ABA−responsive genes, including *ZmRAB18*, *ZmABF2*, and *ZmABI4*, in WT, *ZmSnRK2.9-OE*, and *zmsnrk2.9-crispr* plants under ABA treatment (50 µM). Following ABA treatment, the expression levels of all three genes were markedly higher in the OE lines and lower in the mutants compared with the WT (Figure 5D–F). Hence, these outcomes demonstrated that ZmSnRK2.9 positively regulates the ABA response in maize.

### 2.10. Genetic Relationship of ZmbHLH47 with ZmSnRK2.9

**Figure 6:**
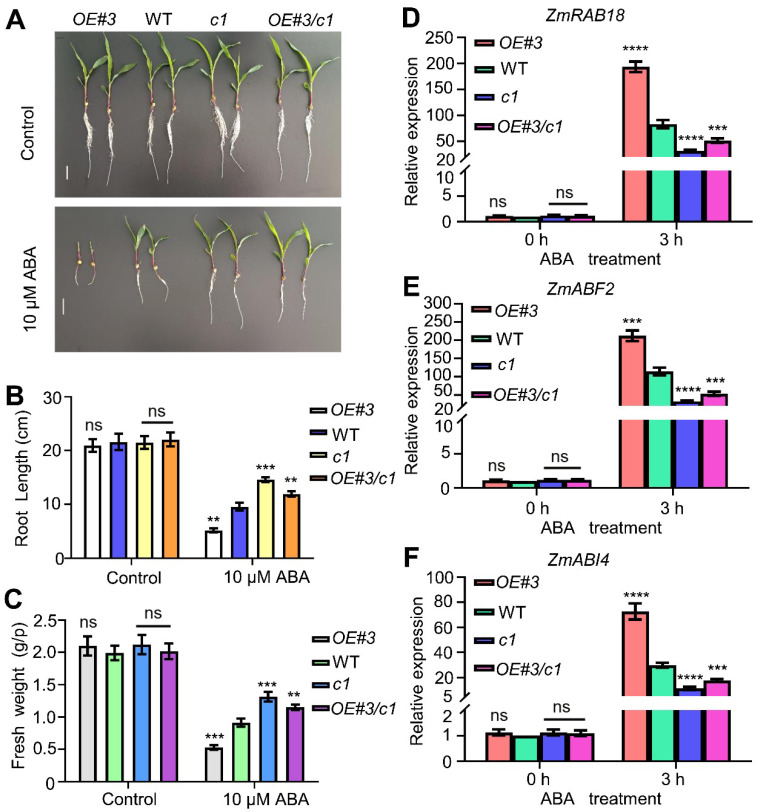
ZmbHLH47 acts upstream of ZmSnRK2.9 to positively the ABA response. (**A**) Growth phenotypes of WT, *ZmbHLH47-OE#3*, *zmsnrk2.9-c1*, and *ZmbHLH47-OE#3 zmsnrk2.9-c1* plants grown in Hoagland solution with 0 or 10 µM ABA. (**B**,**C**) Determination of the root length and fresh weight in (**A**). Data are means of three biological replicates means ± SD (*n* = 60 for (**B**,**C**)). ns indicates no significant difference to the corresponding controls. ** *p* < 0.01; *** *p* < 0.001, indicating significant differences to the corresponding controls (Student’s *t*−test). (**D**–**F**) Analysis of the transcript abundances of ABA–responsive genes in various genotypes with or without 50 µM ABA treatment. Expression in the untreated WT was set to 1.00. Data shown are means ± SD of three biological replicates. ns indicates no significant difference to the corresponding controls. ***, and **** indicate significant difference to the corresponding controls with *p* < 0.001, and *p* < 0.0001, respectively (Student’s *t*−test). In (**A**–**F**), *OE#3* represents *ZmbHLH47-OE#3*, *c1* represents *zmsnrk2.9-c1*, and *OE#3/c1* represents *ZmbHLH47-OE#3 zmsnrk2.9-c1*.

**Figure 7:**
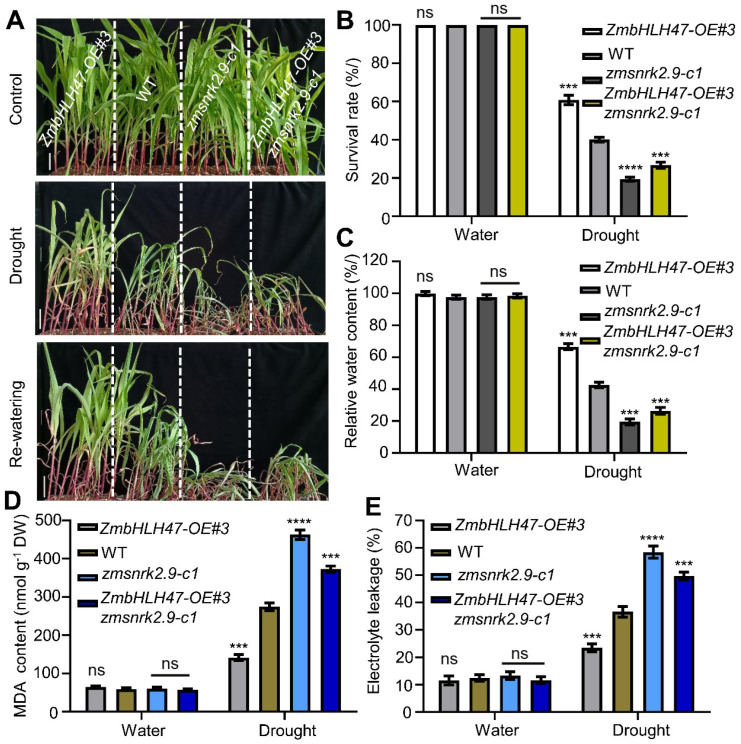
Genetic relationship of ZmbHLH47 with ZmSnRK2.9 in drought tolerance. (**A**) Drought tolerance phenotypes of WT, *ZmbHLH47-OE#3*, *zmsnrk2.9-c1*, and *ZmbHLH47-OE#3 zmsnrk2.9-c1* plants. Scale bars = 5 cm. (**B**) Calculation of survival rate after drought stress in (**A**). At least 30 seedlings of each line per replicate were used for survival rate analysis. The values are presented as means ± SD of three biological replicates. ns indicates no significant difference to the corresponding controls. *** *p* < 0.001; **** *p* < 0.0001, indicating significant differences to the corresponding controls (Student’s *t*−test). (**C**) Relative water content (RWC) of WT, *ZmbHLH47-OE#3*, *zmsnrk2.9-c1*, and *ZmbHLH47-OE#3 zmsnrk2.9-c1* plants under well–watered and drought conditions. Data shown are means ± SD of three biological replicates. ns indicates no significant difference to the corresponding controls. Significant differences are indicated using Student’s *t*−test: *** *p* < 0.001. (**D**,**E**) Malondialdehyde (MDA) content (**D**) and percentage leakage of electrolyte (**E**) of WT, *ZmbHLH47-OE#3*, *zmsnrk2.9-c1*, and *ZmbHLH47-OE#3 zmsnrk2.9-c1* plants under well–watered and drought conditions. DW, dry weight. The values are presented as means ± SD of three biological replicates. ns indicates no significant difference to the corresponding controls. *** *p* < 0.001; **** *p* < 0.0001, indicating significant differences to the corresponding controls (Student’s *t*−test).

To figure out the genetic interplay between ZmbHLH47 and ZmSnRK2.9, we conducted crosses between *ZmbHLH47-OE#3* plants and *zmsnrk2.9-c1* mutants, resulting in the generation of *ZmbHLH47-OE#3 zmsnrk2.9-c1* lines. These lines, alongside *ZmbHLH47-OE#3*, *zmsnrk2.9-c1*, and WT plants, were subjected to ABA and drought stress assays. Under ABA treatment, *ZmbHLH47-OE#3* plants exhibited an elevated ABA sensitivity, while *zmsnrk2.9-c1* plants displayed a reduced sensitivity to ABA compared with the WT plants (Figure 6A–F). *ZmbHLH47-OE#3 zmsnrk2.9-c1* plants predominantly mirrored the behavior of *zmsnrk2.9-c1* plants in terms of root length, fresh weight, and ABA-responsive gene expression (Figure 6A–F). Similarly, following drought stress, *ZmbHLH47-OE#3 zmsnrk2.9-c1* plants closely phenocopied *zmsnrk2.9-c1* plants, as indicated by their drought sensitivity (Figure 7A–E). Cumulatively, these discoveries substantiated the concept that ZmbHLH47 could function as the upstream of ZmSnRK2.9, exerting a positive regulatory influence on ABA response and promoting drought tolerance in maize.

## 3. Discussion

### 3.1. The ZmbHLH47-ZmSnRK2.9 Module Positively Regulates Drought Tolerance in Maize

In recent decades, the escalating threat of drought stress has posed a significant challenge to global agriculture sustainability. Consequently, it has become imperative to elucidate the underlying genetic and molecular mechanisms governing drought resistance in maize for effective crop enhancement. In this investigation, we recognized the ZmbHLH47-ZmSnRK2.9 module as a prospective affirmative modulator of drought tolerance in maize. Initially, ZmbHLH47 was demonstrated to bestow both ABA sensitivity and resilience to drought in maize (Figure 1A–I and Figure 2A–F). Subsequent comprehensive assays revealed that ZmbHLH47 could activate the expression level of the *ZmSnRK2.9* gene, a member of the subgroup III SnRK2 kinases, likely by directly binding to its promoter (Figure S8A–K and Figure 3A–D). Furthermore, the overexpression of *ZmSnRK2.9* heightened ABA sensitivity and drought resistance, while the knockout of *ZmSnRK2.9* in maize diminished ABA sensitivity and drought tolerance (Figure 4A–I and Figure 5A–F). Finally, our genetic evidence suggested that ZmbHLH47 could function as the upstream of ZmSnRK2.9, exerting a positive regulatory influence on ABA response and drought tolerance in maize (Figure 6A–F and Figure 7A–E).

### 3.2. ZmbHLH47 May Specially Respond to Osmotic Stress in Maize

The qRT−PCR analysis revealed a significant upregulation of the *ZmbHLH47* gene in response to NaCl, PEG, and exogenous ABA treatments (Figure S3A–D). Notably, ZmbHLH47 exhibited a specific involvement in ABA signal transduction and drought response in maize, with no discernible role in salt response (Figure 1A–I, Figure 2A–F and Figure S4A–C). It was well−established that drought could induce osmotic stress in plant cells, leading to the rapid accumulation of ABA [6,29]. Conversely, salinity could negatively impact plant growth and development through ionic stress, osmotic stress, and secondary stresses (e.g., oxidative stress) induced by the excess accumulation of reactive oxygen species [61,62,63,64,65]. Consequently, our speculation was that ZmbHLH47 could specifically respond to the osmotic stress triggered by drought in maize. However, a more in−depth understanding of the detailed molecular mechanism necessitates further investigations.

### 3.3. ZmSnRK2.9, One of Subclass III SnRK2s, Functions as a Positive Regulator of ABA Response and Drought Tolerance in Maize

Notably, the subgroup III SnRK2 protein kinases have gained widespread recognition for their pivotal regulatory roles in the ABA signaling pathway and subsequent stress responses in plants [41,66]. In *Arabidopsis*, the well−documented rapid and robust activation of three subclass III SnRK2s—SnRK2.2, SnRK2.3, and SnRK2.6—by ABA could lead to the phosphorylation of downstream effectors, such as AREB/ABFs, significantly contributing to ABA responsiveness and drought tolerance in the plant [67,68]. Similarly, in rice, members of the subclass III group of SnRK2, namely SAPK8, SAPK9, and SAPK10, have exhibited to play crucial roles in controlling ABA–mediated seed germination and seedling growth [69,70]. Despite these insightful findings, the specific contributions of subclass III SnRK2 protein kinases to ABA response and drought tolerance in maize remain to be elucidated. A recent study demonstrated that ZmSnRK2.10, a member of the subgroup III SnRK2 protein kinases, could be activated by ABA, and its overexpression could partially complement the ABA−insensitive phenotype of the *snrk2.2/2.3/2.6* mutant in *Arabidopsis* [41]. In the present investigation, we identified ZmSnRK2.9, another member of the subgroup III SnRK2 kinases, as a positive regulator of ABA response and drought tolerance in maize (Figure 4A–I and Figure 5A–F). Interestingly, in contrast to *Arabidopsis*, where SnRK2.2, SnRK2.3, and SnRK2.6 function redundantly, the *ZmSnRK2.9* single mutant exhibited strong ABA insensitivity and enhanced drought tolerance. We hypothesize that this phenomenon may arise from distinct temporal and spatial expression patterns of subclass III SnRK2 protein kinases in maize. Further evidence is required to validate this hypothesis in the future studies.

## 4. Materials and Methods

### 4.1. Botanical Specimens and Growth Conditions

All plants with overexpressed (OE) genes and those with CRISPR−Cas9−induced mutations were attained from Wimi Biotechnology (Jiangsu) Co., Ltd., situated in Changzhou, China. The respective maize seeds were planted and cultivated in a meticulously controlled growth chamber (Percival). The environment within the chamber was upheld at a 16 h photoperiod featuring 400 µmol m^−2^ s^−1^ light intensity of a temperature of 25 °C, followed by 8 h of darkness at 22 °C. Additionally, a relative humidity was set at 70%.

For the salt treatment, a well–established procedure was followed [71]. Briefly, 2−week−old maize seedlings underwent irrigation with a 200 mM NaCl solution. After an interval of 12 days, comprehensive photographic documentation of the plants was undertaken. Following this, the dry weight of the aerial components of the plants was quantified post–exposure to 200 mM NaCl for a duration of 15 days. Additionally, the survival rate of the plants was intricately calculated after enduring a 35−day treatment with 200 mM NaCl.

The drought treatment protocol was implemented as outlined in [72]. Maize seedlings at the age of 12 days underwent an 8−day water deprivation period, followed by a subsequent 3−day rehydration phase. Phenotypic observations were documented and the survival rate was computed. Relative water content was assessed following the procedure detailed in [73]. About 0.3 g fresh leaf samples (L1–L3 from the top) were collected for malondialdehyde (MDA) content analysis, involving homogenization in 10% trichloroacetic acid (TCA) containing 0.65% 2–thiobarbituric acid (TBA), followed by heating at 100 °C for 15 min, as in [74]. The percentage of electrolyte leakage was assessed following the methodology outlined in [75].

Seedlings at the tri−foliate stage underwent distinct treatments involving immersion in a solution with 200 mM NaCl, 10% PEG 4000, or 50 µM ABA for the designated duration.

### 4.2. Phylogenetic and Sequence Analysis

Utilizing default settings for pairwise and multiple alignments, MEGA 7.0 software was employed to align 13 bHLHs sourced from maize, rice, and *Arabidopsis* [76]. The alignment data were then utilized to construct a phylogenetic tree using the neighbor–joining method [77]. For the analysis of the bHLH domain in ZmbHLH47, the sequence of ZmbHLH47 and its closely related homologs were aligned via Genedoc software (Version 2.7) [78].

### 4.3. Subcellular Localization Assay

For the detailed analysis of the subcellular localization of the ZmbHLH47 protein, we engineered a Super:ZmbHLH47:GFP vector, leveraging the pSuper1300-GFP platform [79]. In this experimental setup, we incorporated the nuclear localization protein D53 (DWARF53) [47]. The co–transformation of maize protoplasts involved the Super:ZmbHLH47:GFP and Super:D53:mCherry vectors. As a negative control, maize protoplasts underwent a genetic modification process involving the co–transformation of Super:GFP and Super:D53:mCherry vectors. The subsequent visualization of fluorescent signals was undertaken utilizing the sophisticated LSM880 laser scanning confocal microscope provided by Carl Zeiss that was headquartered in Jena, Germany. Comprehensive primer details for the subcellular localization assay are summarized in Table S1.

### 4.4. qRT–PCR

In strict accordance with the manufacturer’s protocols, maize tissues underwent a meticulous total RNA extraction process utilizing the FastPure^®®^ Universal Plant Total RNA Isolation Kit (Vazyme, Nanjing, China). Following this, 1 µg of the extracted total RNA underwent a sophisticated reverse transcription procedure using the HiScript^®®^ III RT SuperMix for qPCR (+gDNA wiper) kit provided by Vazyme. The subsequent qRT–PCR was conducted on a state–of–the–art Stratagene Mx3000P real–time system cycler (Agilent) employing the advanced ChamQ Universal SYBR qPCR Master Mix provided by Vazyme. The chosen reference control for this meticulous process was actin1 (GRMZM2G126010). Each experiment was executed with three technical replicates, and a minimum of three independent biological experiments were carried out. Comprehensive details pertaining to the primers for qRT–PCR can be accessible in Table S1.

### 4.5. Y1H Assay

In the DNA binding assay, the promoter region (1.5 kb upstream of ATG) of the *ZmSnRK2.9* gene underwent precise amplification and was seamlessly incorporated into the *pHis2.1* reporter vector. The coding sequence of *ZmbHLH47* was subsequently integrated in−frame with the GAL4 activation domain within the *pGADT7* vector. The Y1H assay unfolded according to the manufacturer’s meticulous instructions (Clontech). The evaluation of ZmbHLH47′s DNA binding activity was predicated on the growth performance exhibited by the co−transformants on selective medium supplemented with 3−Amino−1,2,4–triazole (3−AT). Comprehensive details pertaining to the primers for Y1H assay can be accessible in Table S1.

### 4.6. ChIP−qPCR Analysis

Conducting ChIP−qPCR assay involved intricate steps adhering to established protocols [80,81]. Specifically, four grams of maize seedlings aged three weeks underwent cross-linking in 1% formaldehyde under vacuum, followed by the sonication of the resulting chromatin complexes into fragments ranging from 200 to 500 bp. Immunoprecipitation, washing, and reverse cross−linking were performed using polyclonal anti−Flag M2 beads (Sigma–Aldrich, St. Louis, MO, USA) to capture the protein−DNA complex. The precipitated DNA underwent quantitative PCR analysis, with a minimum of three independent experiments conducted, and the presentation includes one representative dataset. Comprehensive details pertaining to the primers can be accessible in Table S1.

### 4.7. EMSA

EMSA was undertaken following established procedures [82]. The coding sequence of *ZmbHLH47* was inserted into the pET30a vector to produce the His–ZmbHLH47 fusion protein. Subsequently, the purification of the recombinant protein His–ZmbHLH47 was undertaken through Ni Sepharose High Performance (GE Healthcare, Chicago, IL, USA), following the prescribed procedures provided by the manufacturer. It was attempted to carry out EMSA utilizing a Chemiluminescent EMSA kit attained from Beyotime, China. Biotin–labeled probes for EMSA were synthesized by Sangon Biotech Co., Ltd. (Shanghai, China). The details of primers and probe sequences employed in the EMSA are summarized in Table S1.

### 4.8. Transcriptional Activity Assay

To delineate the transcriptional activation domain of ZmbHLH47, there was an attempt to amplify both the intact and truncated sequences of *ZmbHLH47*, followed by incorporating the in−frame with the GAL4 DNA binding domain within the *pGBKT7* vector. These resultant constructs, alongside the *pGBKT7* empty vector, were individually introduced into the yeast strain Y2HGold. The identification of the transcriptional activation domain of ZmbHLH47 was undertaken upon monitoring cell growth on SD/−Trp−His−Ade medium.

To interrogate the transcriptional activation of ZmbHLH47 on *ZmSnRK2.9*, we attempted to conduct co−transfections of effector and reporter constructs in protoplasts isolated from maize seedlings aged two weeks. Using the internal control 35S:REN, the measurement of the activities of LUC and REN was undertaken via the Trans Detect Double–Luciferase Reporter Assay kit provided by TransGen Biotech, utilizing a GloMax 20/20 luminometer attained from Promega for detection. Rigorous experimentation was upheld through a minimum of three independent biological replicates. Comprehensive details regarding primer specifications for the transcriptional activity assay are thoughtfully documented in Table S1.

### 4.9. ABA Content Analysis

Leaves from 12−day−old maize seedlings, subjected to either a 10−day drought treatment or maintained without drought stress, were selected for ABA measurement. About 0.5 g leaves (L1–L3 from the top) were finely ground in liquid nitrogen and then extracted with methanol containing 20% water (*v*/*v*). The purification and quantification of ABA followed the protocol outlined in [83,84]. Three independent biological replicates were conducted, with each biological replicate comprising 10 plants from each line.

### 4.10. ABA Sensitivity Assay

The seeds of diverse genotypes underwent sterilization with 5% sodium hypochlorite for 10 min, followed by thrice rinsing with ultrapure water. Subsequently, the seeds were positioned on wet filter paper for a duration of 4 days until the primary roots attained an approximate length of 3 cm. Afterward, the seedlings were transferred and cultivated in Hoagland solution under greenhouse conditions for 7 days. Representative images were captured, and subsequently, the measurement and calculation of both root length and the fresh weight of each seedling were carried out.

### 4.11. Western Blot Analysis

Total protein extraction from maize seedlings was accomplished using radio immunoprecipitation assay (RIPA) buffer (Solarbio, Beijing, China) supplemented with 1 mM phenylmethylsulfonyl fluoride (PMSF). The ensuing proteins, subsequent to separation through 10% sodium dodecyl sulfate polyacrylamide gel electrophoresis (SDS–PAGE), were translocated onto polyvinylidene fluoride (PVDF) membranes (Millipore, Burlington, MA, USA). These membranes underwent a blocking step in 1× TBST buffer (0.02% Tween in tris–buffered saline (TBS)) supplemented with 5% milk, particularly for 2 h at room temperature. This was followed by an overnight incubation with the primary antibody (4 °C). After extensive washing in 1× TBST buffer, the membranes experienced a 2 h incubation at room temperature with secondary antibodies conjugated to horseradish peroxidase. The detection of blots was undertaken utilizing BeyoECL plus (Beyotime).

### 4.12. Plant Transformation

The coding sequences of *ZmbHLH47* and *ZmSnRK2.9* were amplified and subsequently endowed with a Flag tag at their C termini. Following this, the resulting DNA fragments were integrated downstream of the ubiquitin promoter. The requisite target sites, which were strategically positioned in the inaugural exons of ZmbHLH47 and ZmPYL9 to facilitate the creation of CRISPR/Cas9 knockout lines, were sourced from CRISPR–P (http://crispr.hzau.edu.cn/CRISPR2/) (accessed on 10 June 2021). The acquisition of transgenic plants was executed through the meticulous process of Agrobacterium–mediated transformation. For a more precise exploration, the subsequent analyses were conducted exclusively on the homozygous T4 overexpression (OE) lines. Additionally, in the ensuing experiments, the homozygous CRISPR−Cas9 mutant lines—void of the Cas9 transgene—served as pivotal participants. It is imperative to underscore that this part of the investigation was adroitly executed by Wimi Biotechnology. Comprehensive details pertaining to the primers can be accessed in Table S1.

### 4.13. Statistical Analysis

The examination of datasets comprising two distinct groups entailed a thorough implementation of Student’s *t*−test, where the designation “ns” indicates the absence of a statistically significant difference relative to the corresponding controls. The symbols “*”, “**”, “***”, and “****” were employed to denote a significant difference from the respective controls, with *p*–values falling beneath the thresholds of 0.05, 0.01, 0.001, and 0.0001, respectively. A form of mean ± standard deviation (SD) was regarded to present data. For more intricate comparisons involving multiple groups, an all–encompassing approach was utilized, involving the application of one−way ANOVA followed by Tukey’s test for post hoc analysis. Distinct letters were employed as indicators to denote statistically significant differences (*p* < 0.05) among groups.

### 4.14. Accession Numbers

Sequence data accession numbers are retrievable from the GenBank/EMBL libraries.

ZmbHLH47 (NP_001140536.1, Zm00001d048901); ZmbHLH121 (NP_001152439.1, Zm00001d044216); ZmbHLH104 (NP_001347516.1, Zm00001d054038); ZmbHLH129 (NP_001150072.1, Zm00001d014995); AtPOPEYE (NP_001190030.1, AT3G47640); AtbHLH121 (NP_188620.1, AT3G19860); AtbHLH11 (NP_849566.1, AT4G36060); AtbHLH105 (NP_200279.1, AT5G54680); AtbHLH34 (NP_001327796.1, AT3G23210); OsbHLH63 (NP_001404729.1, Os03g0379300); OsbHLH1 (NP_001409908.1, Os07g0628500); OsILR3 (XP_015626366.1, Os02g0116600); OsbHLH121 (XP_015626280.1, Os02g0433600); ZmSnRK2.9 (NP_001386089.1, Zm00001d033339); ZmActin1 (NP_001148651, Zm00001d010159).

## 5. Conclusions

**Figure 8:**
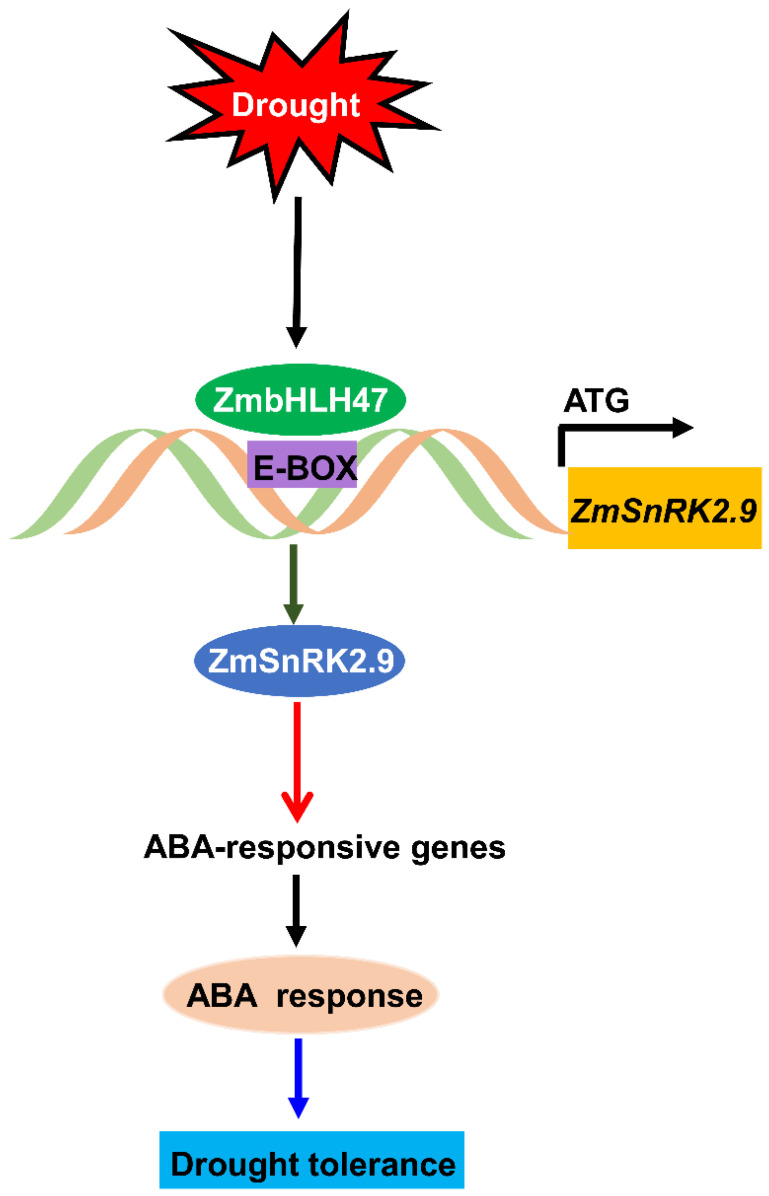
A proposed model for the function of ZmbHLH47-ZmSnRK2.9 module in drought tolerance in maize. Upon environmental drought stress, the transcript abundance and DNA binding affinity of ZmbHLH47 is significantly upregulated, which directly binds to the E−box elements of *ZmSnRK2.9* promoter to activate its expression, leading to the upregulation of ABA−responsive genes. Eventually, the ABA response is promoted, and the drought tolerance is increased in maize. Arrows mean positive regulation.

Drought stress poses a significant threat to global maize production. This study elucidates the role of the ZmbHLH47-ZmSnRK2.9 module in regulating drought tolerance in maize (Figure 8). Under the condition of drought stress, both the transcript abundance and DNA binding affinity of ZmbHLH47 were significantly upregulated. ZmbHLH47 could directly bind to the promoter region of *ZmSnRK2.9*, activating its expression. Consequently, the elevated levels of the ZmSnRK2.9 could contribute to the increased expression levels of ABA response-related genes, ultimately enhancing ABA response and bolstering drought tolerance in maize. This study highlighted the functional dynamics of the ZmbHLH47-ZmSnRK2.9 module in maize’s response to drought, providing valuable insights for the development of drought–resistant crops.

## Data Availability

Data will be made available on request.

## Supplementary Materials

The following supporting information can be downloaded at: https://www.mdpi.com/article/10.3390/ijms25094957/s1.
