# Supplementary material for: The ZmbHLH47-ZmSnRK2.9 Module Promotes Drought Tolerance in Maize
Source: Int J Mol Sci. 2024 May 1;25(9):4957. doi: 10.3390/ijms25094957 (PMC11084430; doi:10.3390/ijms25094957)
Supplement: Supplementary file 1 [file ijms-25-04957-s001.zip › Final Supplementary data.pdf]

The ZmbHLH47-ZmSnRK2.9 Module Promotes Drought Tolerance in Maize

Zhenwei Yan[1], Fajun Zhang[1], Chunhua Mu[1], Changle Ma[2], Guoqi Yao[1], Yue Sun[3], Jing Hou[4], Bingying Leng[1,*], Xia Liu[1,*]

[1]Maize Research Institute, Shandong Academy of Agricultural Sciences, Jinan 250100, Shandong, China; yanzwplant@sina.com (Z.Y.); zhangfj2009@163.com (F.Z.); maizesd@163.com (C.M.); yaoguoqi927@sohu.com (G.Y.)

[2]College of Life Sciences, Shandong Normal University, Jinan 250300, Shandong, China; machangle@sdnu.edu.cn (C.M.)

[3]College of Agronomy, Qingdao Agricultural University, Qingdao 266109, Shandong, China; (sunyue3070601@163.com) (Y.S.)

[4]School of Agriculture, Ludong University, Yantai 264001, Shandong, China; houjing@m.ldu.edu.cn (J. H.)

*Correspondences: Bing-ying Leng (lengbingying@saas.ac.cn); Xia Liu (liuxia@saas.ac.cn)

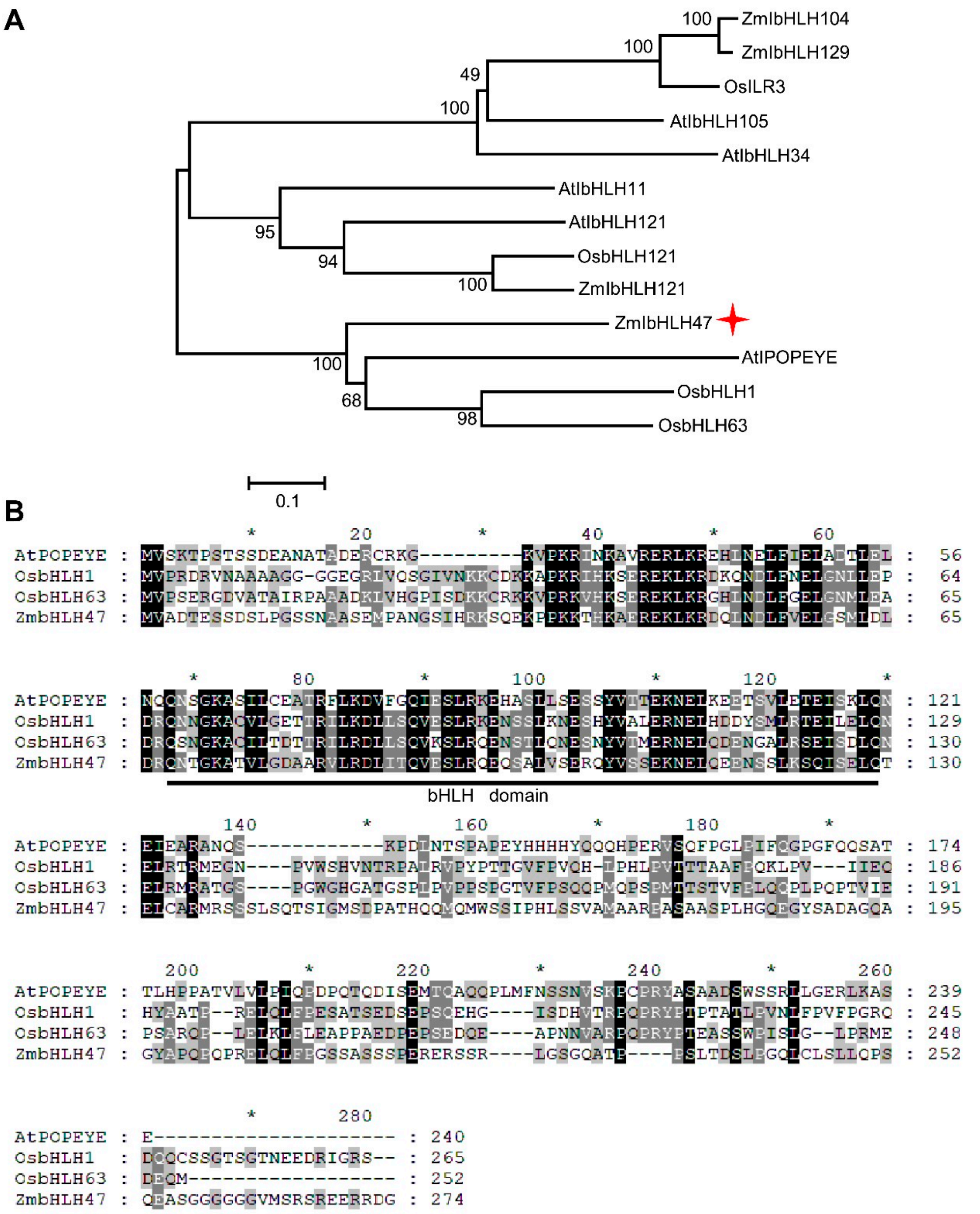

Fig. S1. Phylogenetic and structural feature of ZmbHLH47. (A) Phylogenetic tree analysis of ZmbHLH47 and its closely homologous bHLH proteins from maize, rice, and *Arabidopsis*. Clustal X1.81 program was employed to run sequence alignment, and the phylogenetic tree was constructed by MEGA 7.0. (B) Alignment of ZmbHLH47 with AtPOPEYE, OsbHLH1, and OsbHLH63. The conserved bHLH domain is highlighted with a black line under the alignment.

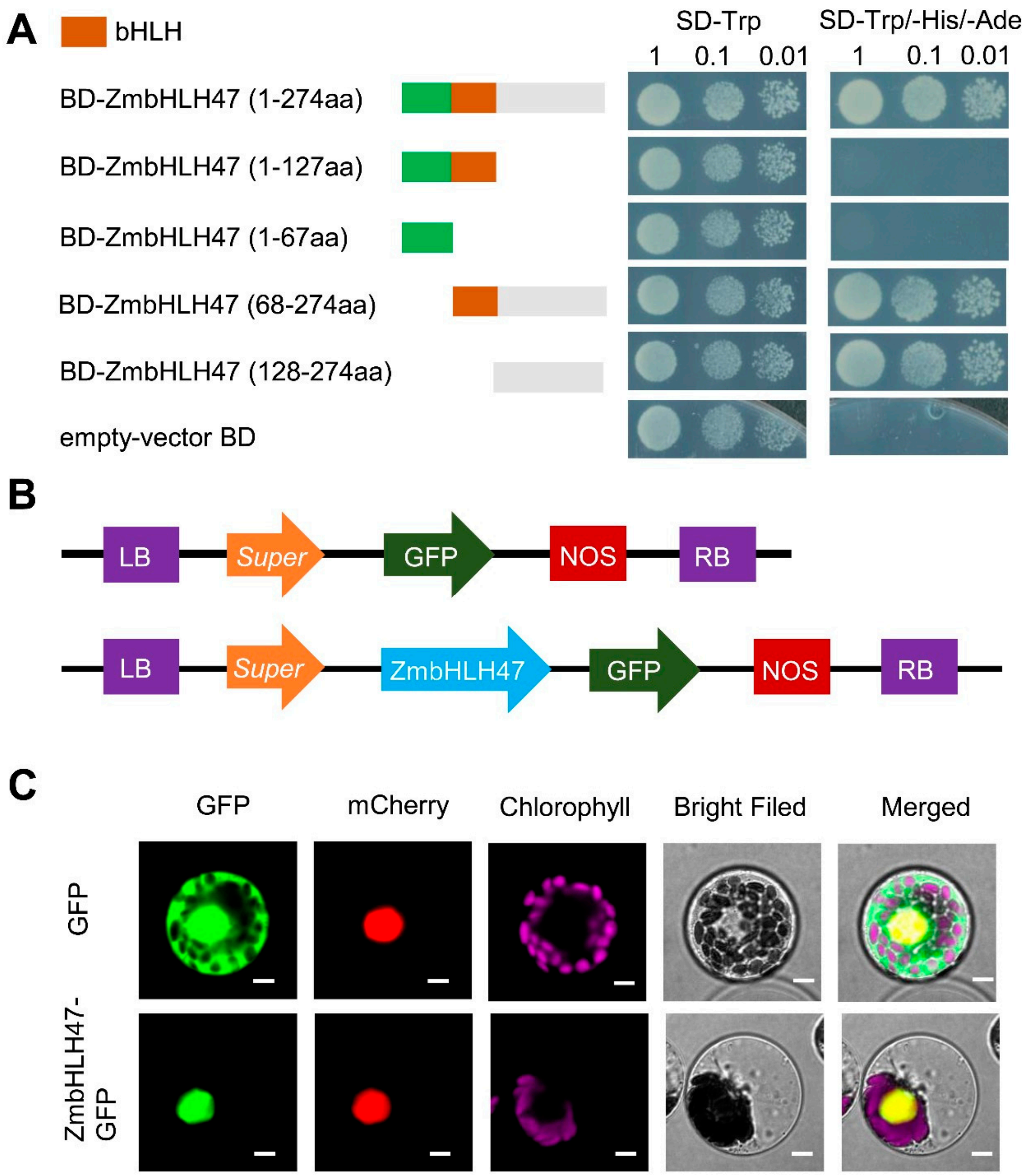

Fig. S2. Transcriptional activation activity and subcellular localization of ZmbHLH47. (A) ZmbHLH47 owns transcriptional activation activity. Full length and truncated mutants of ZmbHLH47 were separately cloned into the *pGBKT7* vector and transformed into yeasts. Yeast cells harboring above-mentioned constructs or *pGBKT7* empty vector (negative control) were plated on SD–Trp or SD–Trp/His/Ade media for 3 days at 28 °C. (B) Schematic diagram illustrating constructs for subcellular localization assay. (C) ZmbHLH47 protein locates in the nucleus. Nuclear localization signal-mCherry indicates a nuclear marker. The scale bar represents 5 μm. Three

independent repeats showed similar results.

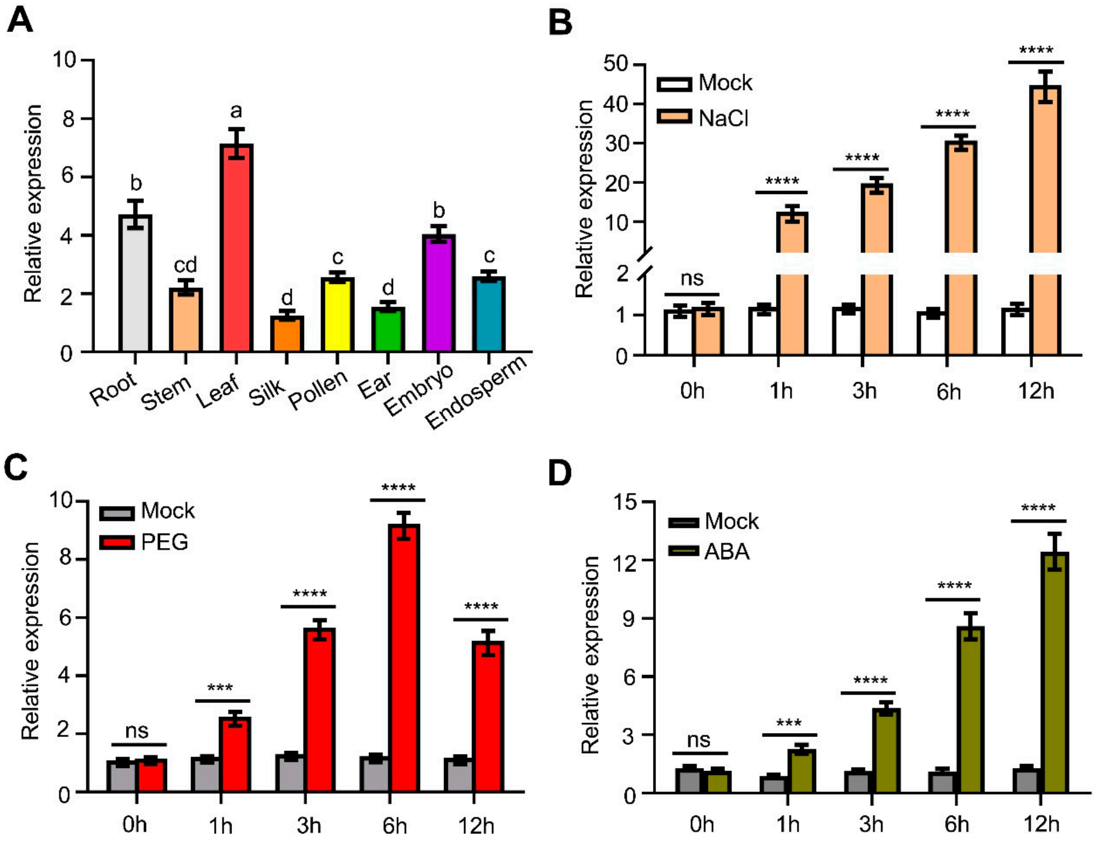

Fig. S3. Expression analysis of *ZmbHLH47* in maize. (A) The determination of the transcript abundance of *ZmbHLH47* in different maize tissues. Root, stem, and leaf samples were taken from plants at the three–leaf stage, silk and pollen samples were taken from plants at the flowering stage, and ear, embryo, and endosperm samples were taken from the maturing stage. Data shown are means ± SD of three biological replicates with one–way ANOVA and Tukey's test. Different letters indicate significant differences ($P < 0.05$). (B–D) qRT–PCR analysis of the expression level of *ZmbHLH47* in response to NaCl, PEG, and ABA treatments. Data shown are means ± SD of three biological replicates. ns indicates no significant difference to the corresponding controls. ***, and **** indicate significant difference to the corresponding controls with $P<0.001$, and $P<0.0001$, respectively (student's *t*–test).

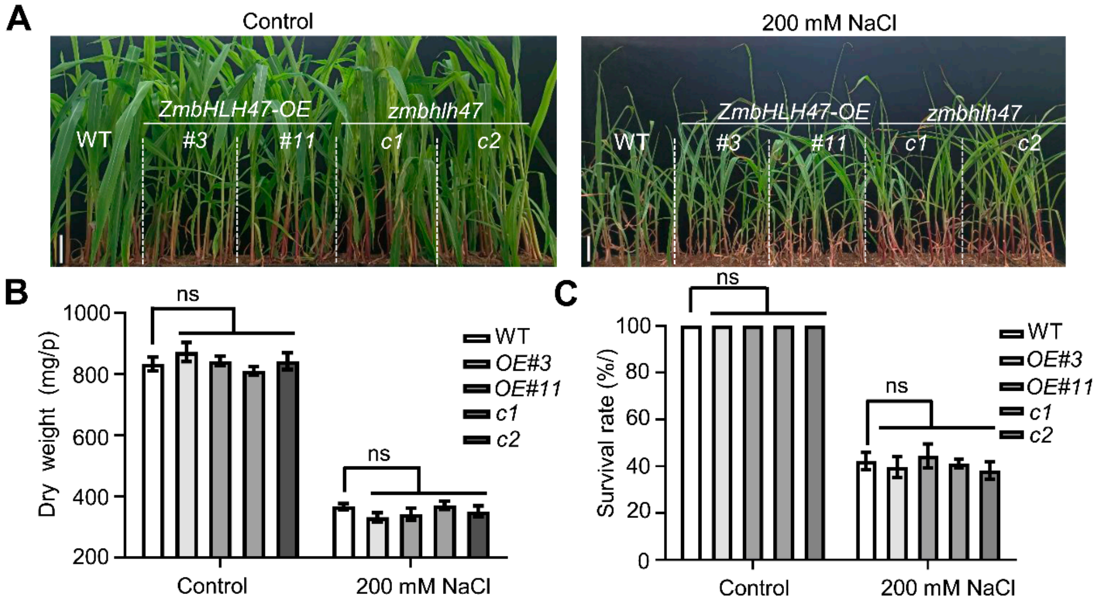

Fig. S4. ZmbHLH47 is not involved in salt stress response in maize. (A) Salt stress phenotype of WT, *ZmbHLH47-OE*, and *zmbhlh47-crispr* seedlings. Scale bars = 5 cm. (B-C) Dry weight and survival rate of WT, *ZmbHLH47-OE* and *zmbhlh47-crispr* seedlings with or without salt stress. At least 30 seedlings of each line per replicate were used for dry weight and survival rate analysis. Data are means of three biological replicates ± SD. ns indicates no significant difference to the corresponding controls (student's *t*-test).

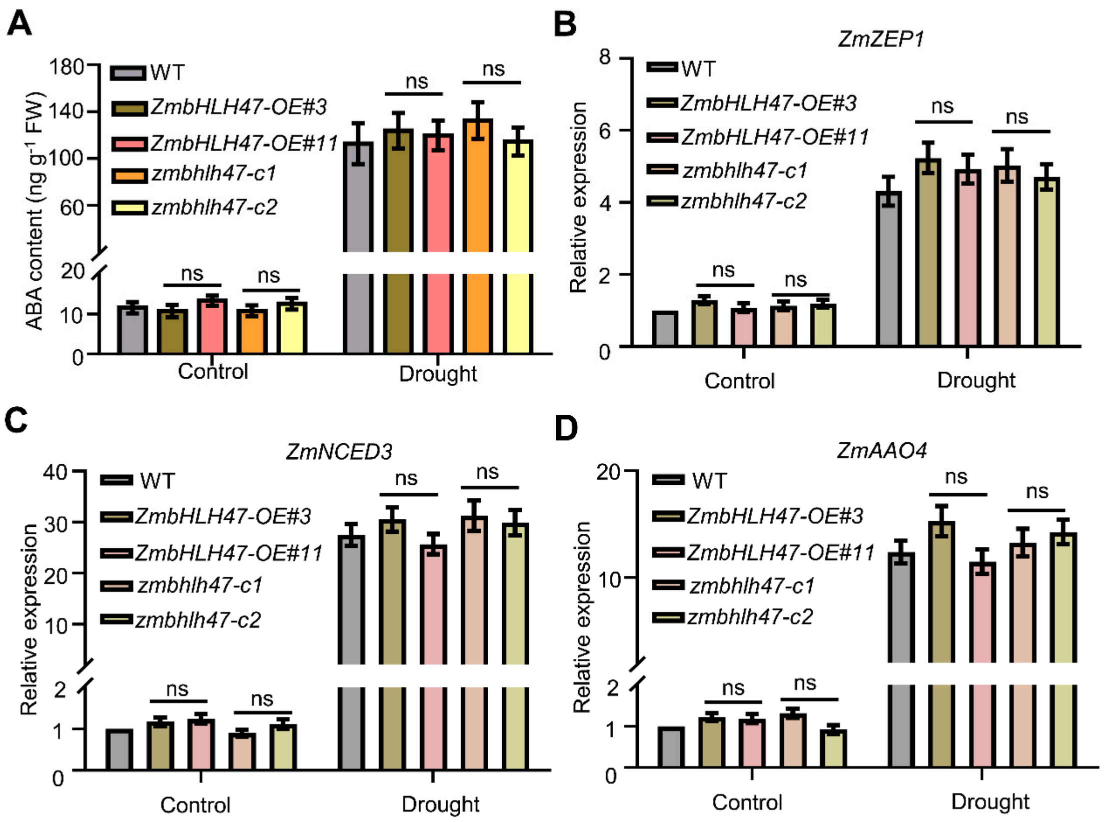

Fig. S5. The content and synthesis of ABA are not influenced by ZmbHLH47. (A) ABA contents were measured in 12–day old WT, *ZmbHLH47-OE*, and *zmbhlh47-crispr* seedlings with sufficient water or drought for 10 d. Data shown are means ± SD of three biological replicates. ns indicates no significant difference to the corresponding controls (student's *t*–test). (B) Expression levels of the key genes involved in ABA biosynthesis in WT, *ZmbHLH47-OE*, and *zmbhlh47-crispr* seedlings under mock and drought stress conditions, as determined by qRT–PCR. Expression in the untreated WT was set to 1.00. Data shown are means ± SD of three biological replicates. ns indicates no significant difference to the corresponding controls (student's *t*–test).

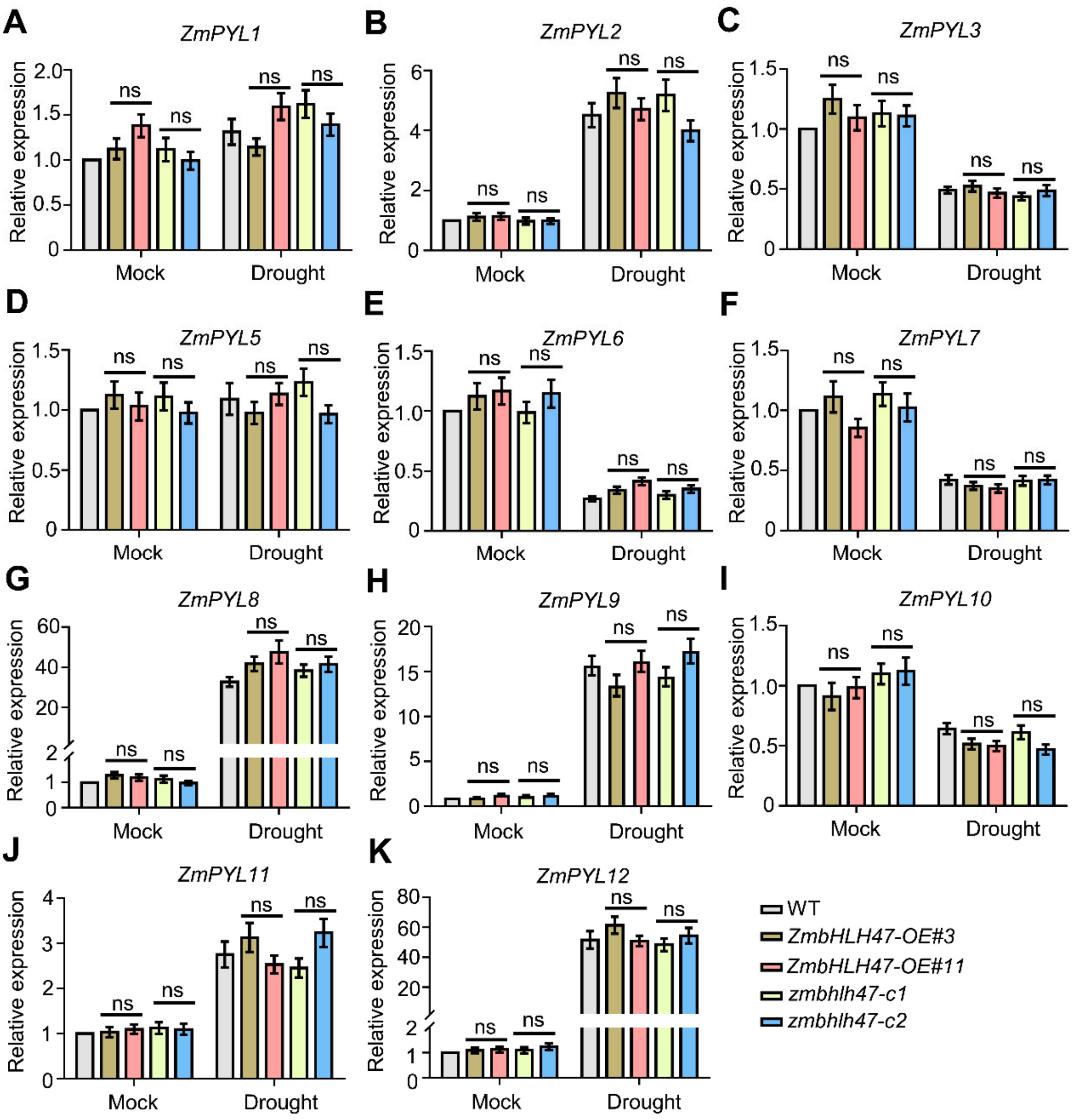

Fig. S6. Expression profiles of *ZmPYL* genes. The transcript abundances of *ZmPYL* genes in WT, *ZmbHLH47-OE*, and *zmbhlh47-crispr* seedlings under mock and drought stress conditions, as determined by qRT–PCR. Expression in the untreated WT was set to 1.00. Data shown are means ± SD of three biological replicates. ns indicates no significant difference to the corresponding controls (student's *t*–test).

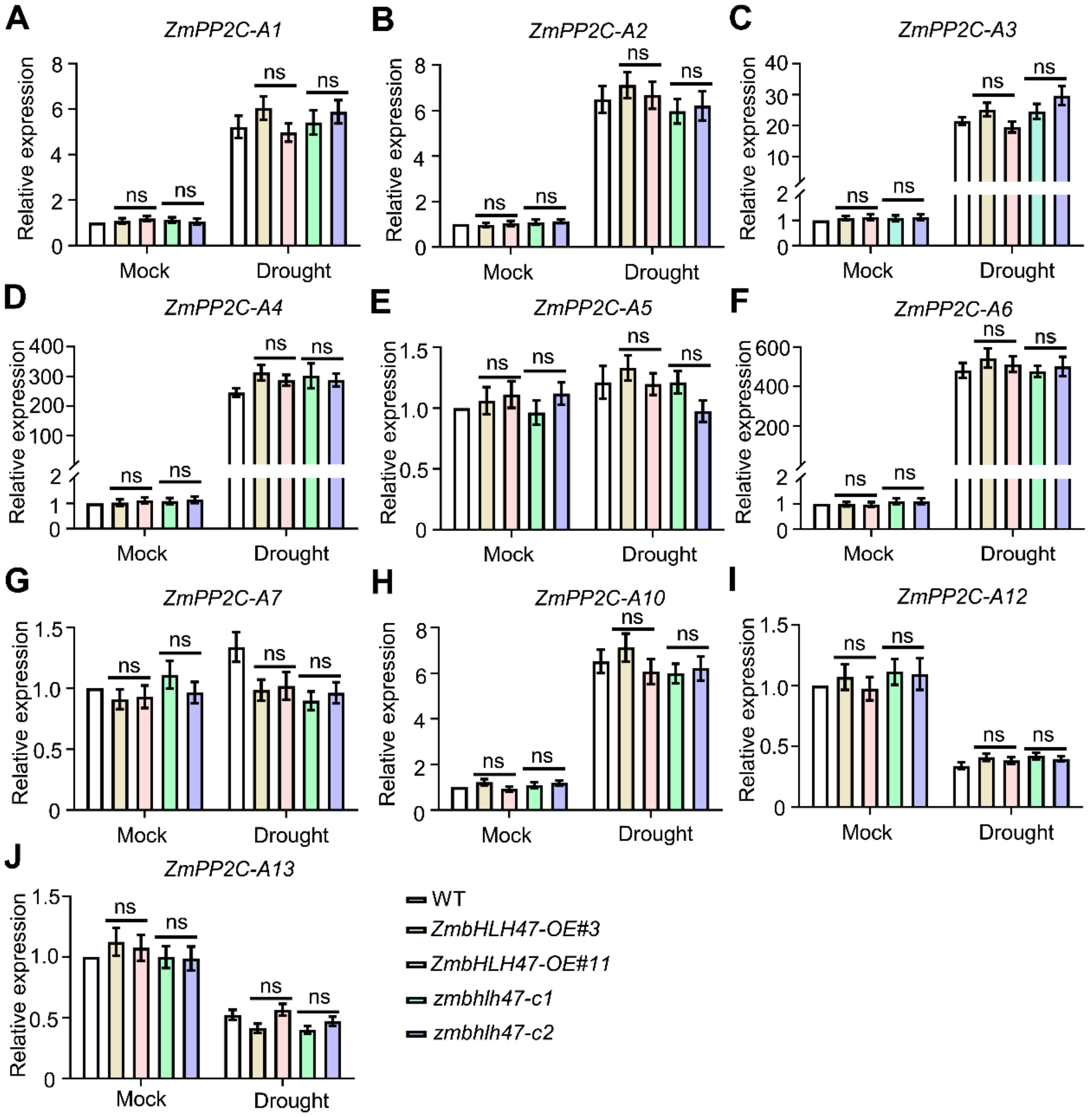

Fig. S7. Expression profiles of *ZmPP2C–A* genes. The transcript abundances of *ZmPP2C–A* genes in WT, *ZmbHLH47-OE*, and *zmbhlh47-crispr* seedlings under mock and drought stress conditions, as determined by qRT–PCR. Expression in the untreated WT was set to 1.00. Data shown are means ± SD of three biological replicates. ns indicates no significant difference to the corresponding controls (student's *t*–test).

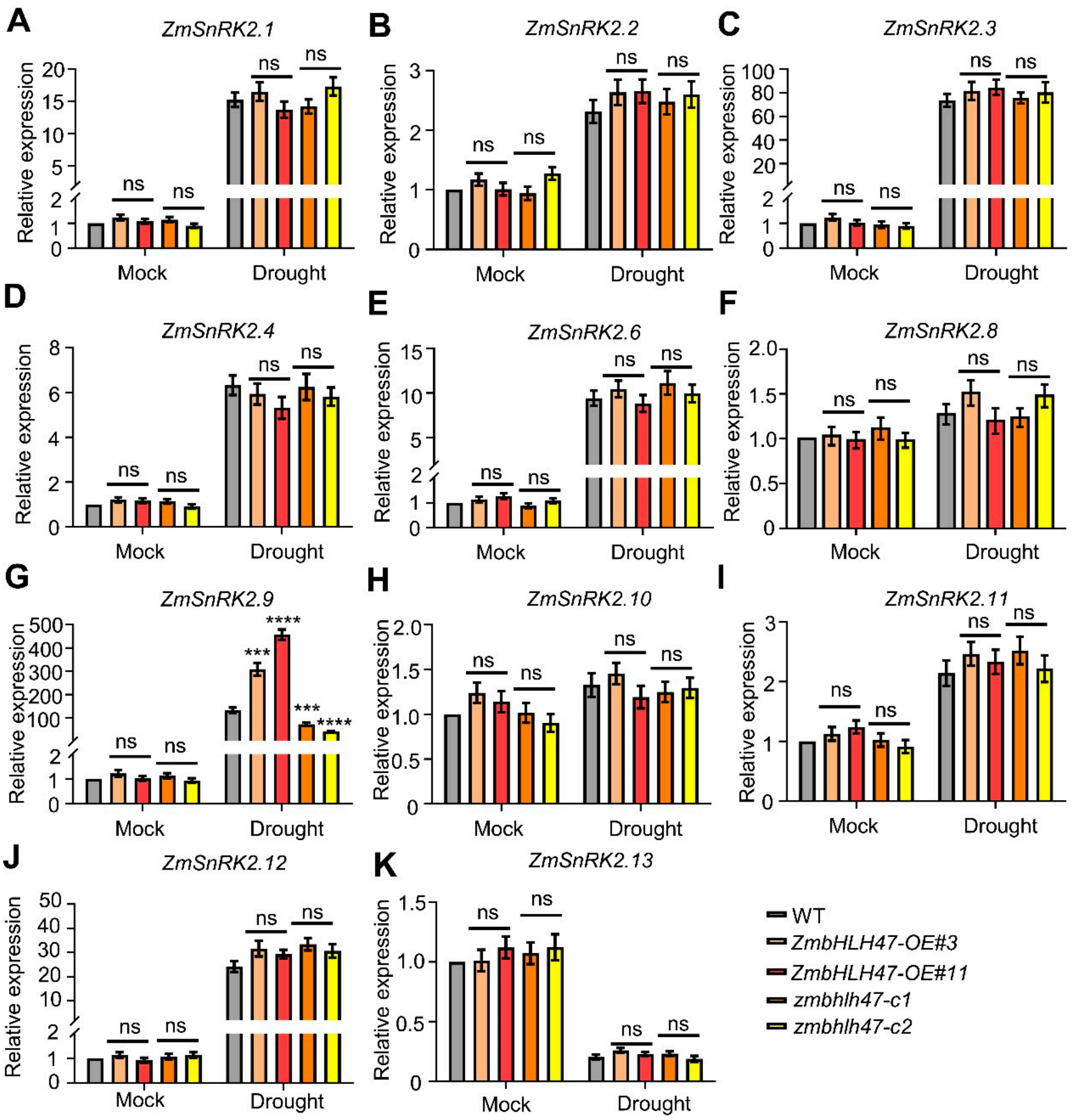

Fig. S8. ZmbHLH47 specially activates the expression of *ZmSnRK2.9* gene under drought stress. The transcript abundances of *ZmSnRK2* genes in WT, *ZmbHLH47-OE*, and *zmbhlh47-crispr* seedlings under mock and drought stress conditions, as determined by qRT–PCR. Expression in the untreated WT was set to 1.00. Data shown are means ± SD of three biological replicates. ns indicates no significant difference to the corresponding controls. ***, and **** indicate significant difference to the

corresponding controls with $P<0.001$, and $P<0.0001$, respectively (student's *t*–test).
